# Spatial learning and long‐term memory impairments in RasGrf1 KO, Pttg1 KO, and double KO mice

**DOI:** 10.1002/brb3.1089

**Published:** 2018-09-26

**Authors:** Lara Manyes, Sarah Holst, Manuel Lozano, Eugenio Santos, Alberto Fernandez‐Medarde

**Affiliations:** ^1^ Lab 1 Cancer Research Center CSIC‐Universidad de Salamanca & CIBERONC Salamanca Spain; ^2^ Laboratory of Food Sciences and Toxicology Faculty of Pharmacy Universitat de València València Spain; ^3^ Department of Neuroscience Karolinska Institutet Stockholm Sweden

**Keywords:** Barnes maze, memory, PCA, Pttg1, RasGrf1, spatial learning

## Abstract

**Background:**

RasGrf1 is a guanine‐nucleotide releasing factor that enhances Ras activity. Human PTTG1 is an oncoprotein found in pituitary tumors and later identified as securin, a protein isolated from yeast with a reported role in chromosome separation. It has been suggested that RasGrf1 is an important upstream component of signal transduction pathways regulating Pttg1 expression and controlling beta cell development and their physiological response. At memory formation level, there are contradictory data regarding the role of RasGrf1, while Pttg1 has not been previously studied. Both proteins are expressed in the mammalian hippocampus, which is one of the key brain areas for spatial learning and memory.

**Objective:**

The aim of this work was to study a potential link between RasGrf1 and Pttg1 in memory formation.

**Method:**

Spatial learning and memory test in the Pttg1 KO, RasGrf1 KO, and Pttg1‐RasGrf1 double KO and their correspondent WT mice using a Barnes maze.

**Results:**

In comparison with the WT control mice, Pttg1 KO mice learned how to solve the task in a less efficient way, suggesting problems in memory consolidation. RasGrf1 KO mice performance was similar to controls, and they learned to use the best searching strategy. Double KO mice reached a better spatial learning level than WT.

**Conclusion:**

A role for Pttg1 in memory consolidation/formation is suggested, while our RasGrf1 KO mice do not show hippocampus associated memory defects.

## 1 INTRODUCTION

Scientific works have revealed that Ras‐GRF proteins are important components of signaling pathways that mediate a variety of central nervous system functions, including hormonal production controlling body size, regulation of synaptic plasticity, and generation of specific forms of learning and memory (Feig, 2011). More specifically, analysis of various knockout mice strains has uncovered a precise functional contribution of RasGrf1 in processes of memory and learning, photoreception, control of postnatal growth and body size, pancreatic β‐cell proliferation and neogenesis, and glucose homeostasis (Fernández‐Medarde & Santos, 2011).

Human PTTG1, an oncogenic protein, has been identified as securin, a protein regulating chromosome separation (Pei & Melmed, 1997; Zou, McGarry, Bernal, & Kirschner, 1999). Mice lacking Pttg1 are viable and fertile but display a sexually dimorphic diabetes mellitus because of defective ß‐cell proliferation (Wang, Moro, Kovacs, Yu, & Melmed, 2003; Wang, Yu, & Melmed, 2001; Yu, Cruz‐Soto, Calzi, Hui, & Melmed, 2006).

Our laboratory showed that pancreatic islets of RasgGrf1 KO mice display a specific transcriptional profile involving significantly reduced expression levels of Pttg1 that are probably linked to a regulatory role of RasGrf1 over the Pttg1 promoter. Furthermore, we have also observed a dominance of Pttg1 over RasGrf1 with regard to the generation of these mouse pancreatic phenotypes, suggesting that RasGrf1 is an important upstream component of signal transduction pathways regulating Pttg1 expression and controlling beta cell development and physiological responses (Manyes et al., 2014).

A possible functional link between these two proteins can also be suggested in retina and hippocampus, where Pttg1 protein expression is repressed in RasGrf1 knock out (KO) mice in comparison with WT controls (Fernández‐Medarde et al., 2007, 2009). The rodent hippocampal system is known to play an important role in spatial learning and memory (Hok, Poucet, Duvelle, Save, & Sargolini, 2016). A high expression of RasgGrf1 is found in this area that has been linked to processes of long‐term memory formation (Barman et al., 2014), although the exact mechanistic contribution of RasGrf1 to these processes remains unclear. On the one hand, it has been published that RasGrf1 KO mice exhibit long‐term potentiation (LTP) defects and associated impairment of amygdale‐dependent learning (Brambilla et al., 1997), whereas more recent studies have rather focused on long‐term depression (LTD) defects and impairment of hippocampus‐dependent learning (Giese et al., 2001; Li, Tian, Hartley, & Feig, 2006).

Pttg1 is expressed in the mouse brain, especially in hippocampus and cerebellum (Lein et al., 2007). This observation, added to the fact that Pttg1 expression is specifically impaired in the hippocampus of RasGrf1 KO mice, prompted us to analyze a potential mechanistic connection between these two proteins in memory formation processes. For this purpose, we tested in this report the spatial learning and memory of Pttg1, Pttg1‐RasGrf1, and RasGrf1 null mice using a Barnes circular maze. Importantly, the protocol used allows measurement of aspects of both reference and working memory within the same test session (Barr, MacLaurin, Semenova, Fish, & Markou, 2007). It is a visuospatial learning and memory task originally designed for rats and subsequently adapted for mice (Malikowska‐Racia, Podkowa, & Sałat, 2018; Sunyer et al., 2007). The Barnes maze is sensitive to impaired hippocampal function and offers advantages as compared to other maze tasks (Sunyer et al., 2007). No food deprivation required and it does not show susceptibility to subtle motor deficits that may be magnified in alternative procedures. It has been posited that the Barnes maze is less stressful to mice than water mazes (Rosenfeld & Ferguson, 2014).

## 2 MATERIALS AND METHODS

### 2.1 Animals

Ninety‐eight C57BL/6J mice, weighing 17.0–34.0 g and between 12 and 14 weeks of age where used. Those included 12 male and 11 female WT and 10 male and 9 female Pttg1 KO; 9 male and 6 female WT and 9 male and 5 female RasGrf1‐Pttg1 KO; 8 male and 8 female WT and 6 male and 5 female RasGrf1 KO mice. Pttg1 KO mice were kindly supplied by Prof. Melmed (Wang et al., 2001). RasGrf1 KO mice were generated as previously described (Font de Mora et al., 2003). WT littermates for each simple KO were generated by crossbreeding simple KO and WT mice. RasGrf1‐Pttg1 double KO mice were generated by crossbreeding simple KO mice and then WT littermates by crossbreeding double KO and WT.

Groups of four animals with the same sex and genotype were housed in type IIL individually ventilated cages, in a temperature‐ and humidity‐controlled room with a 12‐hr light/dark cycle (lights on at 8:00, lights off at 20:00), with food (Teklad 2014, Harlan Laboratories) and water available ad libitum. All testing was completed during the light phase. Animal housing and experimentation followed the general recommendations of the European Communities Council Directive of 2010/63/EU about the use of experimental animals with scientific aims. Maximal efforts were made to minimize the total number of animals used as well as their suffering. The experimental procedures were approved by the local Animal Ethics Committee of the University of Salamanca.

### 2.2 Handling

Animals were handled daily for a period of 5 days during the pre‐experimental period to reduce variations in the behavioral test. The week before starting the test handling was performed. Every day at the same hour each mouse was handled during one minute to reduce stress during the behavioral test. The handling was executed by the same researcher who was also going to conduct the behavioral experiment.

### 2.3 Visual discrimination test

As the performance of the Barnes maze test needs visual cues to be recognized, a visual discrimination test was applied to check mice visual function. The 98 animals were tested after the last handling session of each mouse. As described by Fernández‐Medarde et al. (Fernández‐Medarde et al., 2009), RasGrf1 KO mice may show difficulties to carry out the task because of their retinal photoreception defects. In contrast, retinas of Pttg1 KO mice up to 9 months of age have a healthy morphology and normal photoreceptor function (Yetemian & Craft, 2011).

**Figure 1:**
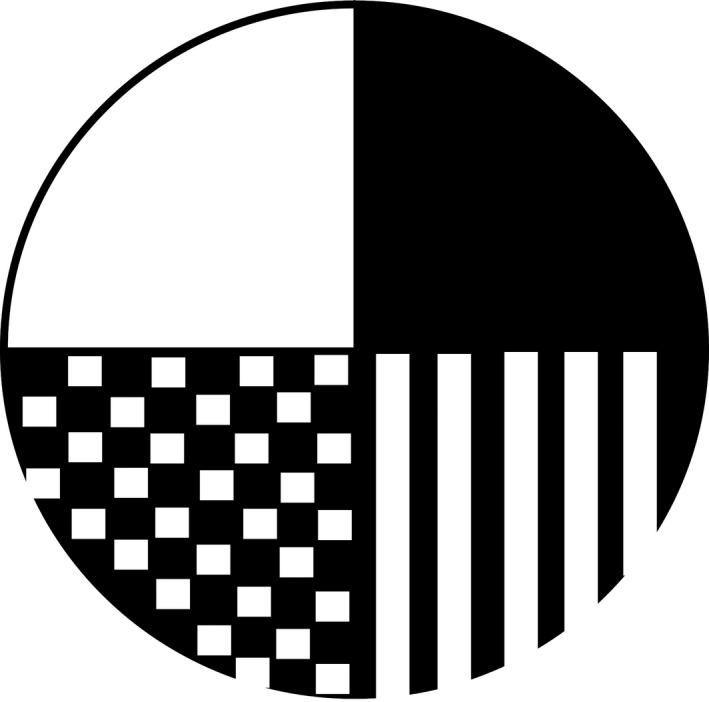
Plastic cover with four different areas adapted to the Barnes maze platform to analyze the mice visual discrimination capacity

A plastic cover with four different areas (white, stripped, checked, and black) adapted to the Barnes maze platform (Figure 1). Each animal was placed for 5′ in the four divided area table. Mice were placed one by one in the middle of the platform covered with an opaque box for 10 s. Then, lights were turned on (3,000 lux) and the mouse was released to explore the table during 5 min. The % of time spent on each area was measured and how the animal passed from one area to the next. The amount of time spent in each area was measured in seconds and determined individually for each mouse and session by the researcher during mouse performance. The seconds were translated into %, being the total time spent on the plastic cover equal to 100%.

### 2.4 Behavioral experiment

**Figure 2:**
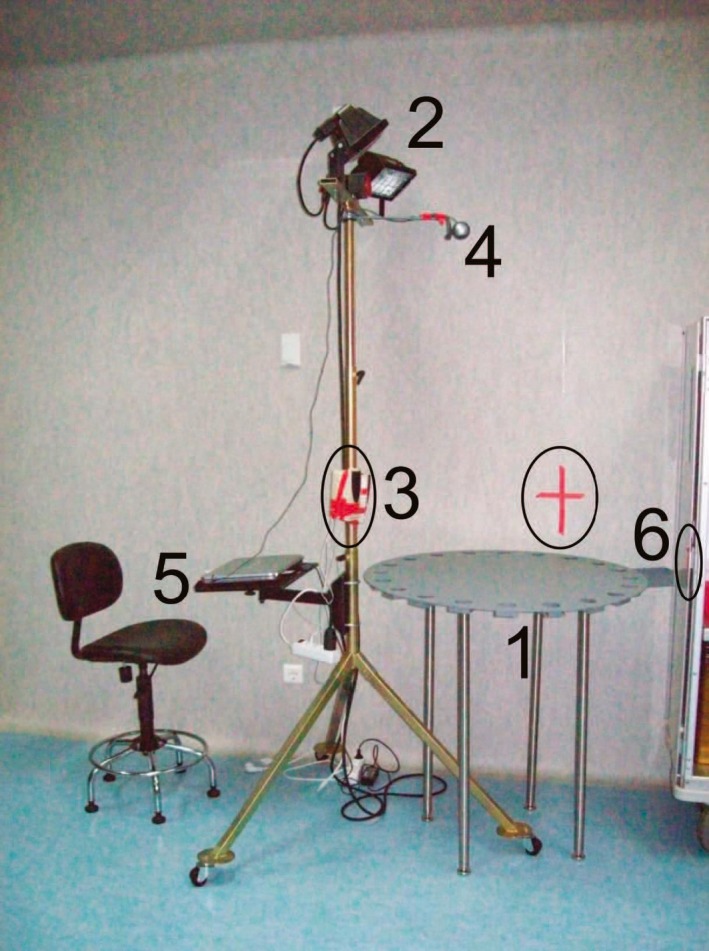
Barnes maze installation. 1. Barnes maze Table 2. Lights at the required high to achieve 3000 lux (lumen/m^2^) on the Table 3. Speakers to apply 80 dB white noise during sessions. 4. Webcam. 5. Researcher location during sessions. 6. Scape tunnel (open), during each session it rests unseen under the table

Learning and memory was evaluated as described by Barr, MacLaurin, Semenova, Fish & Markou (2007). The Barnes maze setup is shown in Figure 2. The Barnes maze was a grey nonreflective circular base plate (91 cm diameter) with 20 holes (5 cm) and an escape box under a hole (Stoelting Europe). As cues, red tape was used (Figure 2). Cue density was low to moderate, consistent with previous rodent studies (Sawada et al., 2005). To reduce intra‐maze odor cues, the maze surface and all the holes were cleaned with 70% ethanol between each session.

Briefly, each mouse was tested during 42 days, dividing the data collection in four phases (Table 1). The task yields measures of the parameters which are presented in Table 2. From the combination of timepoint and parameter abbreviations the variable names are obtained. Working memory errors were those in which the mouse returned to a hole it had previously explored. Perseverations represented repeated sequential explorations of a hole (other than the target) or two adjacent holes. Search strategies for each mouse's daily session were classified as the percentage of time spent using one of three defined categories: (a) random search strategy: localized hole searches separated by crossings through the center; (b) serial search strategy: systematic hole searches in clockwise or counterclockwise direction; or (c) spatial search strategy: reaching the escape tunnel with both error and distance scores of less than or equal to 3. The amount of time spent using each search strategy was measured in seconds and determined individually for each mouse and session by the researcher using the video recordings. The seconds were translated into %, being the total time to scape equal to 100%.

**Table 1:** Barnes maze protocol phases and data collection timepoints

Days	Phase	Abbreviation of timepoint
1–4	Training 1 with data acquisition	T1
5–18	Training without data acquisition	
19–22	Training 2 with data acquisition	T2
23–34	12‐day break without testing	
35–38	Memory retention with data acquisition	MR
39–42	Reverse learning (escape hole 180º from its initial position) with data acquisition	RL

**Table 2:** Parameters collected on each session

Parameters	Abbreviation	Unit
Latency to investigate the first hole	t1H	s
Working memory errors	WE	*n*
Perseverations	P	*n*
Distance from the first hole explored to the target hole (inclusive)	X.1H	*n*
Total number of holes explored	nH	*n*
Total time to escape	t	s
Search strategy: Random	RS	%
Serial	SERS	%
Spatial	SPAS	%

Variable abbreviations in Tables 3 and 5 are a combination of one parameter (Table 2) and one timepoint (Table 1).

**Table 3:** Friedman test on the selected parameters with genotype gender as factor

Group	Total time			Working errors			Number of holes explored			Spatial search strategy		
	χ^2^ statitics	*df*	*p*‐value	χ^2^ statitics	*df*	*p*‐value	χ^2^ statitics	*df*	*p*‐value	χ^2^ statitics	*df*	*p*‐value
Pttg1.WT+male	21.30	3	0.000091	17.24	3	0.000632	7.10	3	0.068700	10.52	3	0.014600
Pttg1.WT+female	22.32	3	0.000056	22.56	3	0.000050	23.25	3	0.000036	16.29	3	0.000991
Pttg1.KO+male	15.71	3	0.001303	15.22	3	0.001640	21.18	3	0.000096	9.00	3	0.029291
Pttg1.KO+female	15.28	3	0.001591	11.10	3	0.011216	9.00	3	0.029291	4.98	3	0.173268
RasGrf1‐Pttg1.WT+male	8.92	3	0.030318	22.78	3	0.000045	15.74	3	0.001281	9.00	3	0.029291
RasGrf1‐Pttg1.WT +female	21.38	3	0.000088	25.38	3	0.000013	22.59	3	0.000049	15.29	3	0.001584
RasGrf1‐Pttg1.KO+male	12.60	3	0.005587	17.73	3	0.000501	9.36	3	0.024845	12.00	3	0.007383
RasGrf1‐Pttg1.KO +female	11.16	3	0.010891	11.87	3	0.007844	8.28	3	0.040566	10.36	3	0.015762
RasGrf1.WT+male	3.96	3	0.266197	8.92	3	0.030404	7.24	3	0.064629	0.08	3	0.994007
RasGrf1.WT+female	10.80	3	0.012858	12.75	3	0.005210	4.93	3	0.176829	5.13	3	0.162870
RasGrf1.KO+male	16.20	3	0.001032	9.58	3	0.022461	12.83	3	0.005011	15.25	3	0.001615
RasGrf1.KO+female	12.06	3	0.007172	7.67	3	0.053427	12.31	3	0.006405	11.73	3	0.008378

### 2.5 Statistical analysis

In the visual discrimination test, KO mice results were compared to the correspondent WT using *T*‐test.

For Barnes maze results, principal component analysis (PCA) was performed to select the most significant variables that explain the data variability. The median of the data from four different timepoints (phases) was used in the selected set. An index is defined for each variable *X*_i_ that measures its contribution to all principal components in which the variable is involved. We define this contribution index (CI) as a weighted mean of its contribution to each component with the explained variance of the components as weight. This is,

$${\mathrm{CI}}_{i}=\sum_{k=1}^{r}c_{k_{i}}v_{k},$$

where *r* is the number of components, $c_{k_{i}}$ is the contribution to the component *k*, and *v*_k_ is the explained variance for this component. The values of $c_{k_{i}}$ are provided by PCA. The variables with highest contribution are chosen, and the CI represents a balance between the loss of information and an effective reduction in the number of variables.

The data were analyzed for normality by assessing the sample distribution or skewness (−1.5 to +1.5 considered normally distributed). Data were analyzed by Friedman's ANOVA, with genotype sex as a between‐subjects factor and time as a within‐subject factor. Wilcoxon signed‐rank test was used as post hoc test. Kruskal–Wallis test was used to analyze significant differences between groups during a timepoint. A significance level of *p* < 0.05 was regarded as statistically significant. All analyses were performed with SPSS program (SPSS, http://scicrunch.org/resolver/SCR_002865RRID:SCR_002865).

## 3 RESULTS

### 3.1 Visual discrimination test

**Figure 3:**
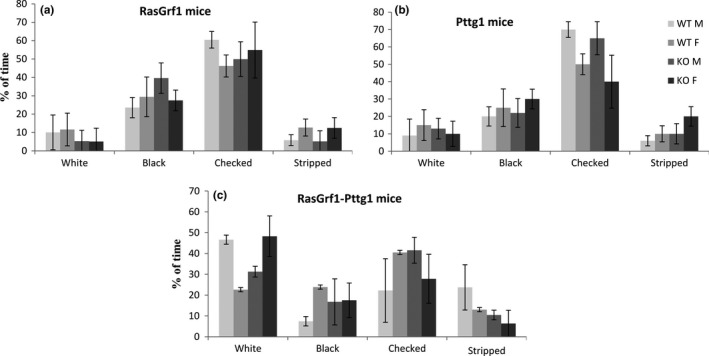
Bar graph representing the time (% of time) spent in each area (types of area: white, black, checked, and stripped) by the mice depending on the genotype during the visual discrimination test. Error bars represent the SEM

In order to be sure that the mice had no visual problems, mice went through an easy experiment of black and white visual discrimination. All mice showed a clear preference for the total black and/or the black and white checker areas (Figure 3). When they explored the limit between two areas for the first time, all of them were cautious and tried to cross slowly. No differences in the performance between neither mice phenotypes nor sex were observed.

### 3.2 Principal component analysis

In order to start the analysis of the large amount of data recorded during the Barnes maze test, it is critical to use statistics that include multivariate analysis to select the most significant among them. This way it is possible to reduce the set of variables to explain the data variability. To do this it is used (PCA), as a first step, although not in the classical way to interpret the principal components but to select a subset of variables. The aim is getting the most relevant variables considering the contribution of each variable to each principal component, through a contribution index; hence, the number of variables is reduced (Lozano et al., 2018).

#### 3.2.1 Contribution index

The final selection of the variables took into account the CI obtained based on the studied mice group (Pttg1, Pttg1‐RasGrf1, and Rasgrf1). These CI are standardized between 0 and 1, and the addition of three, named *total* CI*,* is 3 at the most. The variables that obtained the highest scores in every mice group analyzed were those related to the total time (t_MR total CI = 2.7422 and t_T2 total CI = 2.7149), the working memory errors (WE_T1 total CI = 2.8578 and WE_MR total CI = 2.7365), the number of holes explored (nH_MR total CI = 2.8661; nH_T2 total CI = 2.7996; nH_RL total CI = 2.7638; and nH_T1 total CI = 2.7270), and the spatial search strategy (SPAS_T2 total CI = 2.8873 and SPAS_MR *total* CI = 2.6609).

Total time needed to finish the task gives information of the learning level achieved and its quality. Working memory errors count the number of times that a mouse explores holes already explored. More working errors lead to longer session and worst search strategy. Spatial search strategy is the most efficient search strategy mice can use in Barnes maze. The less efficient strategy is random search, which was only used in T1 although some mice returned to it in RL. The best performance would be a small amount of total time, no working errors, a number of holes explored equal to or lower than 3, and 100% of time applying spatial search strategy.

#### 3.2.2 Total time

No significant differences were found between the WT and the KO RasGrf1 or RasGrf1‐Pttg1 males and females (Table 4). Total time spent by Pttg1 mice showed significant differences between periods for each group (Table 3), but also between groups in T2 (Table 4).

**Table 4:** Kruskal**–**Wallis test with the categories of the selected variables as factors

Variable	Pttg1			Pttg1‐RasGrf1			RasGrf1		
	χ^2^	gl	*p*‐value	χ^2^	gl	*p*‐value	χ^2^	gl	*p*‐value
t_T1	7.363	3	0.061	1.821	3	0.610	0.153	3	0.985
t_T2	8.840	3	**0.031**	5.711	3	0.127	4.002	3	0.261
t_MR	4.219	3	0.239	5.877	3	0.118	2.432	3	0.488
t_RL	1.504	3	0.681	4.506	3	0.212	1.088	3	0.780
WE_T1	8.619	3	**0.035**	1.510	3	0.680	1.320	3	0.724
WE_T2	2.266	3	0.519	5.385	3	0.146	6.746	3	0.080
WE_MR	4.599	3	0.204	1.545	3	0.672	5.668	3	0.129
WE_RL	1.978	3	0.577	1.473	3	0.688	1.188	3	0.756
nH_T1	7.321	3	0.062	4.825	3	0.185	0.985	3	0.805
nH_T2	2.944	3	0.400	7.342	3	0.062	3.187	3	0.364
nH_MR	0.575	3	0.902	5.290	3	0.152	5.116	3	0.163
nH_RL	12.834	3	**0.005**	4.951	3	0.175	7.624	3	0.054
SPAS_T1	5.122	3	0.163	7.160	3	0.067	0.497	3	0.920
SPAS_T2	6.377	3	0.095	9.024	3	**0.029**	6.294	3	0.098
SPAS_MR	0.765	3	0.858	6.488	3	0.090	5.868	3	0.118
SPAS_RL	8.035	3	**0.045**	1.510	3	0.680	4.278	3	0.233

Note

Bold values highlight statistically significant *p* value.

**Figure 4:**
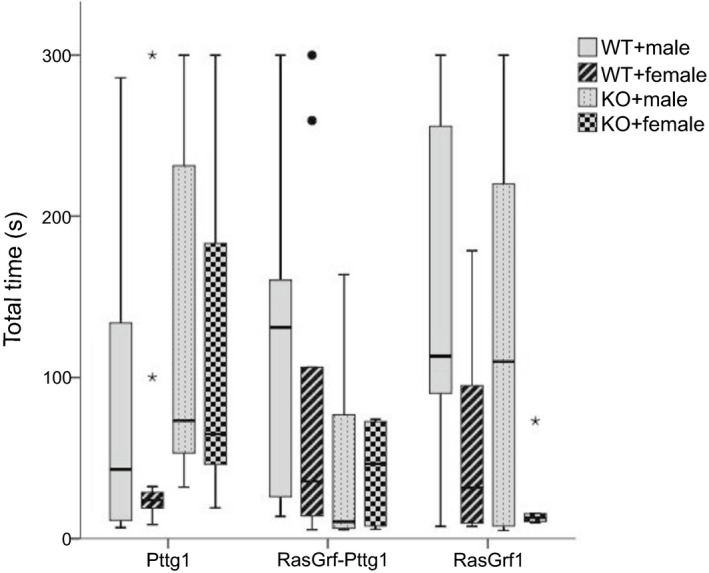
Boxplot representing time invested (seconds) by the genetically modified mice groups (RasGrf1, RasGrf1‐Pttg1, and Pttg1) to finish the task during timepoint T2

In Figure 4, it can be observed that Pttg1 WT groups used less amount of time to finish the task than Pttg1 KO groups, especially females. Only Pttg1 groups showed significant differences between phases in the same mice group (Table 5).

**Table 5:** Homogeneous groups obtained from Wilcoxon signed‐rank test used as post hoc test

Group	Total time	Working errors	Number of holes explored	Spatial search strategy
	Homogeneous groups	Homogeneous groups	Homogeneous groups	Homogeneous groups
Pttg1.WT+male	**T1≠(T2,MR,RL)**; T2 = MR; T2 = RL; **MR≠RL**	T1 = T2 = RL;**T1≠MR**;T2 = MR=RL	T2 = T1;**T1≠MR**;(T1,T2)=RL;**MR≠RL**	T1 = T2 = MR=RL
Pttg1.WT+female		**T1≠(T2,MR,RL)**; T2 = MR=RL	**T2≠T1**;**T1≠(MR,RL)**;T2 = MR;**T2≠RL**;**MR≠RL**	
Pttg1.KO+male	**T1≠T2**;T1 = MR=RL;T2 = MR=RL	**T1≠(T2,MR)**;T1 = RL;T2 = MR=RL	**T2≠T1**;**T1≠(MR,RL)**;T2 = (MR,RL);MR=RL	
Pttg1.KO+female	T1 = T2 = RL;**T1≠MR**;T2 = MR=RL	T1 = T2 = MR=RL	T1 = T2 = MR=RL	
RasGrf1‐Pttg1.WT+male	T1 = T2 = MR=RL	**T1≠(T2,MR)**;T1 = RL;T2 = MR=RL	**T2≠T1**;**T1≠(MR,RL)**;T2 = (MR,RL);**MR≠RL**	
RasGrf1‐Pttg1.WT+female		**T1≠(T2,MR)**;T1 = RL;T2 = MR=RL	**T2≠T1**;**T1≠MR**;T1 = RL;T2 = MR;**T2≠RL**;**MR≠RL**	
RasGrf1‐Pttg1.KO+male		T1 = T2 = MR=RL	**T2≠T1**;**T1≠(MR,RL)**;T2 = MR;**T2≠RL**;MR=RL	
RasGrf1‐Pttg1.KO+female			T2 = T1;**T1≠MR**;T1 = RL;**T2≠(MR,RL)**;**MR≠RL**	
RasGrf1.WT+male			T1 = T2 = MR=RL	
RasGrf1.WT+female				
RasGrf1.KO+male			**T2≠T1**;**T1≠MR**;T1 = RL;T2 = MR; **T2≠RL**;**MR≠RL**	
RasGrf1.KO+female				

Note

Bold values highlight statistically significant *p* value.

#### 3.2.3 Working memory errors

No significant differences were found between the WT and the KO RasGrf1 or RasGrf1‐Pttg1 males and females in Kruskal**–**Wallis test. Pttg1 mice showed significant differences between groups in T1, but at this initial phase of the behavioral experiment, it cannot be interpreted as a defect in learning (Table 4). Three Pttg1 and the two RasGrf1 WT mice groups showed significant differences between T1 and other phases in the same mice group (Table 5). The data showed a general decrease in all mice of the number of WE made during the development of the task. No significant differences were found between the WT and the KO RasGrf1 or RasGrf1‐Pttg1 males and females.

#### 3.2.4 Number of holes explored

Wilcoxon test showed that almost every group of mice performed differently in each phase (Table 5). As in the two previous parameters analyzed, no significant differences were found between the WT and the KO RasGrf1 or RasGrf1‐Pttg1males and females. By contrast, Pttg1 mice showed significant differences between groups in RL (Table 4).

**Figure 5:**
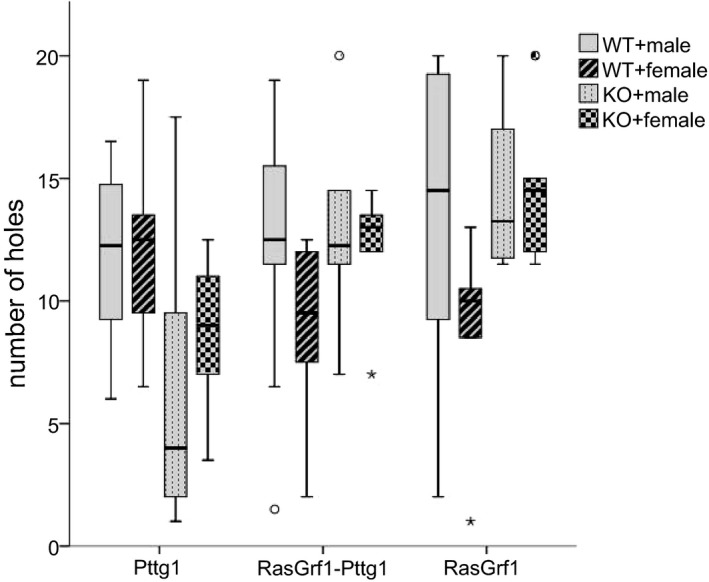
Boxplot representing number of holes explored (number of holes) by the mice genotypes tested (RasGrf1, RasGrf1‐Pttg1, and Pttg1) during the task in timepoint RL

In Figure 5, it can be observed that Pttg1 KO groups explored fewer holes than WT in RL phase. A deep analysis of the performance of these mice pointed out that KO groups never learned where the escape hole was, so when turning it 180º degrees from its initial position, these KO mice kept their number of holes explored unchanged while WT groups augmented the number.

#### 3.2.5 Spatial search strategy

This parameter presented opposed results than the test measuring the number of holes explored, and no significant differences were found between phases in any mice group (Table 5). Nevertheless, Pttg1 null mice showed differences in this parameter at RL and RasGrf1‐Pttg1 at T2 (Table 4).

**Figure 6:**
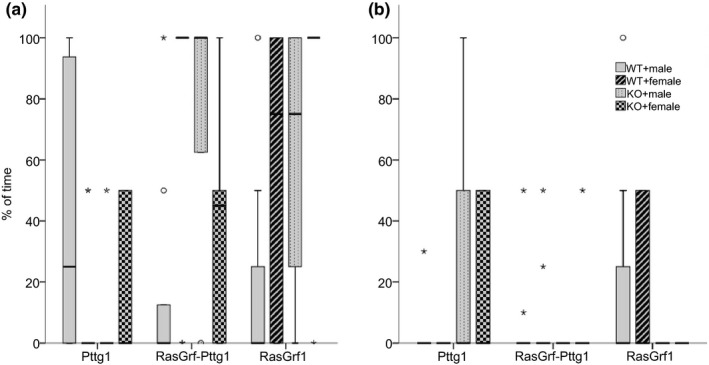
Boxplot representing the amount of time (%) that spatial search strategy is used by the different genotypes tested (RasGrf1, RasGrf1‐Pttg1, and Pttg1) during the task in T2 (a) and RL (b) timepoints

In Figure 6a, it can be perceived that all mice groups used spatial search strategy but the significant differences in RasGrf1 mice groups show that KO groups used more this strategy than WT, pointing towards a better spatial learning. Results in Figure 6b are consistent with Figure 4, Pttg1 KO groups used more spatial strategy than WT, so a smaller number of holes were explored even if they never memorized where the hole was.

## 4 DISCUSSION

Our data regarding RasGrf1 KO mice match those of d'Isa et al. (2011), which after years of controversy, demonstrated that RasGrf1 KO mice have no spatial memory defects (Table 6), but impaired contextual fear conditioning. In particular, the report of d'Isa et al. (2011). used the GENA53 mouse line, in which a nonsense mutation was introduced in the RasGrf1 coding region without additional changes in the genome and explained the different spatial learning phenotype found between Giese et al. (2001) and Brambilla et al. (1997) mouse lines, as potentially attributable to genomic alterations produced as a consequence of the different position of the mutation within the RasGrf1 locus or the insertion of the neomycin resistance cassette. It is pertinent to mention in this regard that the targeting strategy used for our KO mice strains is similar to that used by Giese et al. (2001) or Brambilla et al. (1997) but the use of a less stressing/fear producing methodology may account for the discrepancies with the data published by those groups and the similarity of our results to those of d'Isa and co‐workers (d'Isa et al., 2011).

**Table 6:** Summary of the results obtained using the Barnes maze

Group	Total time	Working errors	Number of holes explored	Spatial search strategy
Pttg1.WT versus Pttg1.KO	Significant differences after training (T2): KO mice, independently of sex, needed more time to finish the task (if they did finish) than the WT.	Significant differences before training (T1): KO mice made more mistakes than WT during the first sessions.	Significant differences in reverse learning (RL): As WT mice learned the task they kept looking for escape hole in the other side of the maze.	Significant differences in reverse learning (RL): Pttg1 KO groups used more spatial strategy than WT in this timepoint because they did not learnt the task.
RasGrf1.WT versus RasGrf1.KO	No differences	No differences	No differences	No differences
RasGrf1‐Pttg1.WT versus RasGrf1‐Pttg1.KO	No differences	No differences	No differences	Significant differences after training (T2): KO male mice used more than WT the most effective search strategy but not females.

Regarding the Pttg1 mice (Table 6), it should be taken into account that a relation between insulin and spatial learning was previously described (Muller et al., 2011) and it was also established that starved, 3‐month‐old Pttg1 KO male mice, but not Pttg1 KO female mice, have a 50% reduction in insulin levels (Wang et al., 2003). Furthermore, blockade of NMDA receptors leads to impairment of neuronal plasticity (Collingridge & Bliss, 1995) and it is also known that insulin‐dependent phosphorylation of NR2A and NR2B subunits modulates NMDA activation (Christie, Wenthold, & Monaghan, 1999). This difference between Pttg1 KO males and females (that show an increased insulin sensibility) could account, at least in part, for the differences of task performance observed in the Barnes maze between Pttg1 KO male and female groups (Wang et al., 2003).

Regarding their performance in the Barnes maze (Table 6), RasGrf1‐Pttg1 double KO mice and RasGrf1 single KO mice behaved in a similar way. Females from both genotypes, especially the RasGrf1 KO ones, learned better how to resolve the task and, in general, both KO groups learned to resolve it equally to or better than their WT counterparts. Our observations documented that RasGrf1 elimination is predominant over Pttg1 depletion regarding the generation of the effects observed on hippocampus spatial function in RasGrf1‐Pttg1 KO mice. A plausible explanation is that the transcriptomic changes linked to RasGrf1 depletion dominate over the changes due to Pttg1 deletion and somehow compensate the effect of Pttg1 elimination in the hippocampus. In any event, our observations indicate that a functional relationship exists between these two proteins regarding this organ's development or synaptic plasticity.

Pttg1 single KO mice showed impaired spatial behavior compared to WT, suggesting a defect in one or more hippocampal functions. The depletion of Pttg1 could be causing developmental problems in this organ or, alternatively, the absence of some of the Pttg1 noncanonical functions might be causing synaptic plasticity defects leading to impaired spatial memory.
